# PROMOTING MENTAL HEALTH LITERACY AMONG OLDER ADULTS IN THE EAST: AN INTEGRATIVE FRAMEWORK

**DOI:** 10.1093/geroni/igad104.3756

**Published:** 2023-12-21

**Authors:** Jessie Ho-Yin Yau, Hotinpo Sky Kanagawa, Maggie Wai-Shan Lo, Stella Lai-Kuen Wong, Gloria Hoi-Yan Wong, Terry Lum, Tianyin Liu

**Affiliations:** The University of Hong Kong, Hong Kong, Hong Kong; The University of Hong Kong, Hong Kong, Hong Kong; The University of Hong Kong, Hong Kong, Hong Kong; The University of Hong Kong, Hong Kong, Hong Kong; The University of Hong Kong, Hong Kong, Hong Kong; The University of Hong Kong, Hong Kong, Hong Kong; The Hong Kong Polytechnic University, Hong Kong, Hong Kong

## Abstract

Although mental health literacy is of considerable importance to advance the welfare of older adults, previous research has shown that the public has a poor understanding of mental health and recognition of mental health symptoms. Traditional mental health literacy promotion programmes utilized a top-down approach; no bottom-up model had been used. Moreover, the existing participatory research paradigms were developed in the West and might not be applicable in the East, given different socioeconomic and cultural contexts. This research aimed to fill the gap by co-creating an integrative framework with stakeholders to promote mental health literacy among older adults in the East using a community-based participatory approach as a case illustration. We conducted a mental health literacy promotion pilot project in five Hong Kong districts from May 2021 to August 2023, spanning over 2 years. Each district formed an action committee, which comprised 5-10 community older adults and 2 social workers, and 2 researchers. Each district had its own promotional activities initiated and designed by older adults, including street booths, art workshops, and publicity materials to promote mental health and introduce relevant information and resources. We conducted six focus groups (n=25) with stakeholders, and we recorded our experiences on reflection notes. Collected data suggested specific elements are important for an integrative framework in the East, including participant empowerment, stakeholders’ role and expectation management, and potential community contribution. We advance gerontological research and practice by providing an integrative framework to promote mental health literacy in the East. Implications will be discussed further.

